# Supplementary material for: Hyperbranched Polyglycerols as Robust Up-Conversion Nanoparticle Coating Layer for Feasible Cell Imaging
Source: Polymers (Basel). 2020 Nov 4;12(11):2592. doi: 10.3390/polym12112592 (PMC7694285; doi:10.3390/polym12112592)
Supplement: Supplementary file 1 [file polymers-12-02592-s001.pdf]

polymers

Supplementary of Hyperbranched polyglycerols as robust up-conversion nanoparticle coating layer for feasible cell imaging

Mingcong Hao †, Tongtong Wu †, Qunzhi Chen, Xueyan Lian, Haigang Wu * and Bingyang Shi *

School of Life Sciences, Henan University, Kaifeng 475003, Henan Province, China; haomc@vip.henu.edu.cn (M.H.); 1014753190785@vip.henu.edu.cn (T.W.); cqz723902674@163.com (Q.C.); 2434246004@qq.com (X.L.)

† These authors are equal.

* Correspondence: whg_2018@henu.edu.cn (H.W.); bs@henu.edu.cn (B.S.)

Received: 4 October 2020; Accepted: 29 October 2020; Published: date

Experiment Part

Chemical reagents and materials

$YCl_3 \cdot 6H_2O$, $YbCl_3 \cdot 6H_2O$, $ErCl_3 \cdot 6H_2O$ and $GdCl_3 \cdot 6H_2O$ were purchased from Aladin company with 99.9% metal basis. Glycidol (anhydrous), oleic acid, 1-octadecene and diaethylamine (>95%) were purchased from J&K company (Shanghai, China). The common reagnts, for example NaOH, NH4F, cyclohexane, ethanol and methanol, were purchased from the Lingfeng Chemical company (Shanghai, China).

Preparation of GFP protein:

Plasmid transformation: Added 2ul *pET-28b-GFP*, the recombinational plasmid with GFP DNA sequence, into 50 uL *E.coli* (BL21-DE3) turbid liquid followed by the incubation in an ice box for 30 min in a tube. Then transferred the tube into water bath with 42 ℃ for 90 s and put it back to the ice box for another 2 min. Incubated the turbid liquid under 37 ℃ with 180 rpm after adding 400uL LB liquid medium, coating the turbid liquid on the LB solid medium. Finally, incubated the solid medium under 37 ℃ overnight.

Collect thalli of *E.coli*: Pick the single colony on the solid medium and enlarged the cultivation in 5 mL LB liquid medium overnight. Then added them into 100 mL fresh LB liquid medium for another 3 hours until the OD/260 of the turbid liquid reached 0.8. Incubated them for another 12 hours under 16 ℃ with 180 rpm. Finally, collected the thalli via centrifugation of 5000 rpm under 4 ℃.

Purification GFP: Sonicated the collected thalli for 20 min followed by freeze thawing 3 times. Collected the suspension and induced the prepared suspension flowed through the as-balanced Ni-HisTrap FF column several times. Then washed the column with imidazole of various concentrations and collected the effluent liquid by 2ml tubes.

SDS-PAGE assay: Measured the purity of GFP via SDS-PAGE.

Hemolysis assay: The hemolysis assay was measured according to the method as reported: Red blood cell (RBC) suspension (0.1 mL, 16%) was added into 5 mL of PBS that contained guanidine-hbPGs/GL3 or PEI/GL3 at the different final concentrations (0.005, 0.05, 0.5 and 5 mg/mL, respectively). PBS and distilled water were, respectively, used as the negative and positive controls. All samples were incubated for 4 h. Then, the suspensions were centrifuged at 1000 rpm for 5 min, and the absorbance values of the released hemoglobin were tested at 540 nm with a microplate reader. The ex-periment was performed three times. The hemolysis was calculated as the formula:

$$\text{hemolysis } (\%) = (A-C)/(B-C) \times 100 \quad (1)$$

A, B, and C, respectively, represent the absorbance of guanidine-hbPGs/GL3 or PEI/GL3, the positive control, and the negative control.

Fabrication of oleic acid capped up-conversion nanoparticles (UCNPs, $NaYF_4$:18%Yb^{3+}/4%Er^{3+}/14% Gd^{3+}, $NaYF_4$:Yb,Er, Gd): Synthesis of UCNPs was performed by using thermal decomposition method with modified protocol (Liu et al. Nature, ***2017***, 543(7644): 229-233.). Firstly, $YCl_3·6H_2O$ (1.28 mmol), $YbCl_3·6H_2O$ (0.36 mmol), $ErCl_3·6H_2O$ (0.08 mmol) and $GdCl_3·6H_2O$ (0.28 mmol) were dissolved into 4 mL of de-ionized water, then aqueous solution was added into 45 mL of mixed solution (15 mL oleic acid and 30 mL 1-octadecene). This mixed solution was kept in an oil bath with 120 ℃ for 30 min, and then nitrogen gas was bubbled to completely remove water in 156 ℃ oil bath for another 60 min. After reaction finished and cooled to room temperature, 10 mL methanol solution containing NaOH (5.0 mmol) and NH_4F (8.0 mmol) were added to the crude products with vigorously stirring for 30 min. Then, the methanol was removed by bubbling nitrogen gas at 50 ℃ for 30 min. After that, solution with organic solvent was heated to 290 ℃ for 1.5 h under nitrogen ambient. UCNPs powder was obtained by adding 20 mL of ethanol into the final solution and washed for several times with cyclohexane and ethanol.

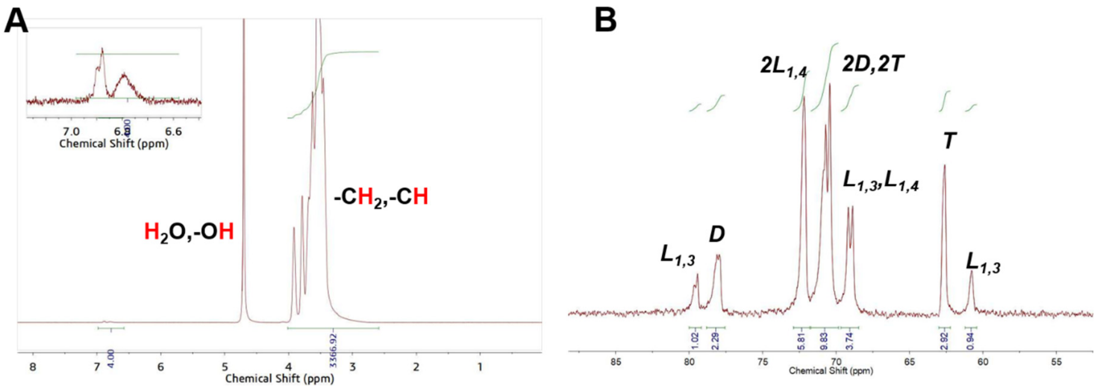

Figure S1. A. 1H NMR spectrum of hbPGs in D_2O (400 MHz). **B**. Inverse gated ^{13}C NMR spectrum of hbPGs in D_2O (400 MHz).

Table S1. The abundance of repeating units in hbPGs.

**Units**	***D***	***T***	***$L_{1,3}$***	***$L_{1,4}$***
Ratio (%)	25.28	32.23	10.38	32.11

Based on the average molecular weight of units and the total amount of repeating units (3366.92/4=673.384).

The number-based molecular weight:

$$M_n=(25.28\%*65.07+32.23\%*83.09+82.08*(10.38\%+32.11\%))*673.384=67.75 \text{ kDa}. \quad (2)$$

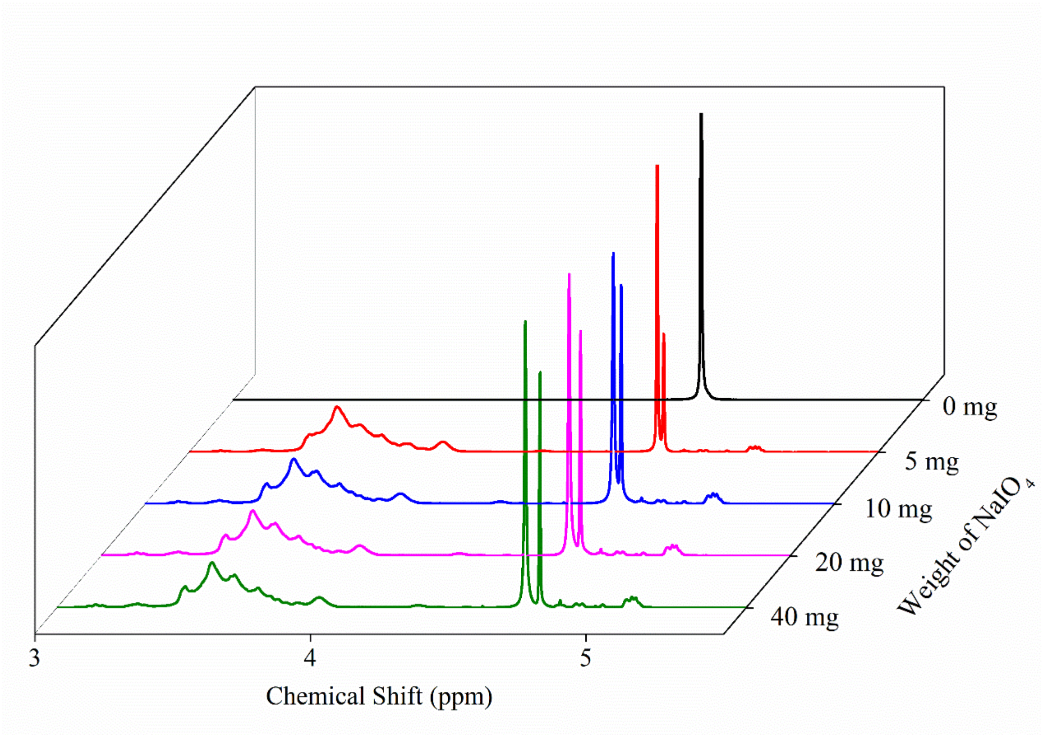

Figure S2. Overlay spectra of hbPGs within $NaIO_4$ titrating in D_2O.

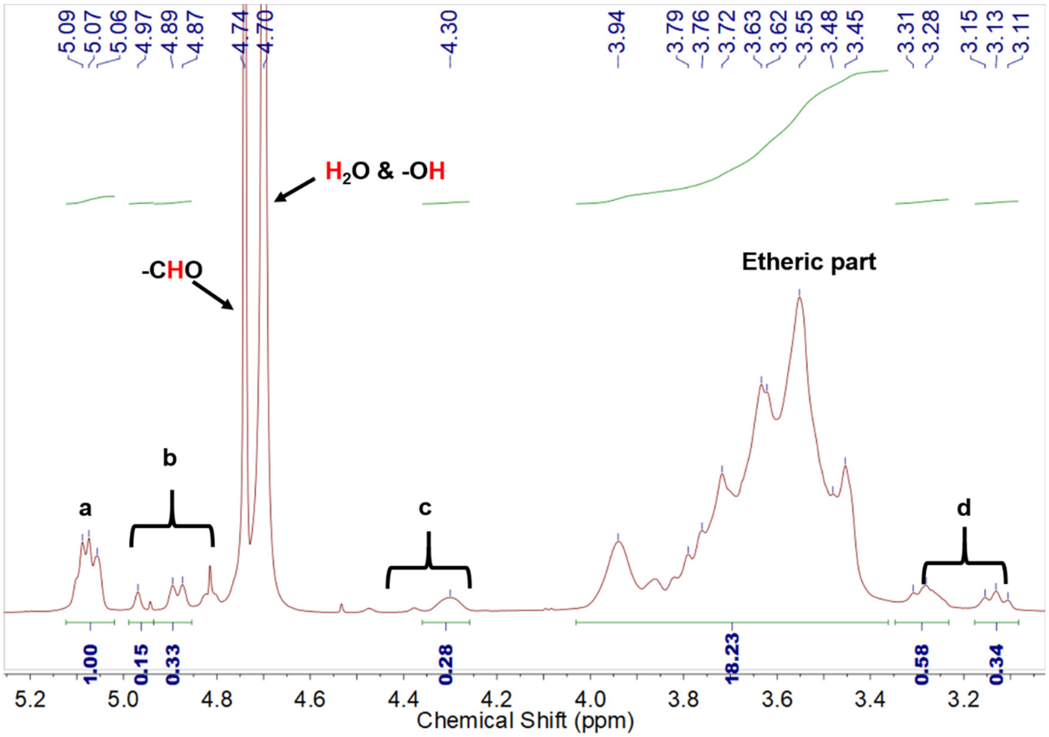

Figure S3. ^{1}H NMR spectrum and calculus information of aldehyde-terminated hbPGs (hbPGs reacted with 20 mg $NaIO_4$) in D_2O. The ratio between peak (5.08 ppm) and etheric skeleton is 18.23:1.

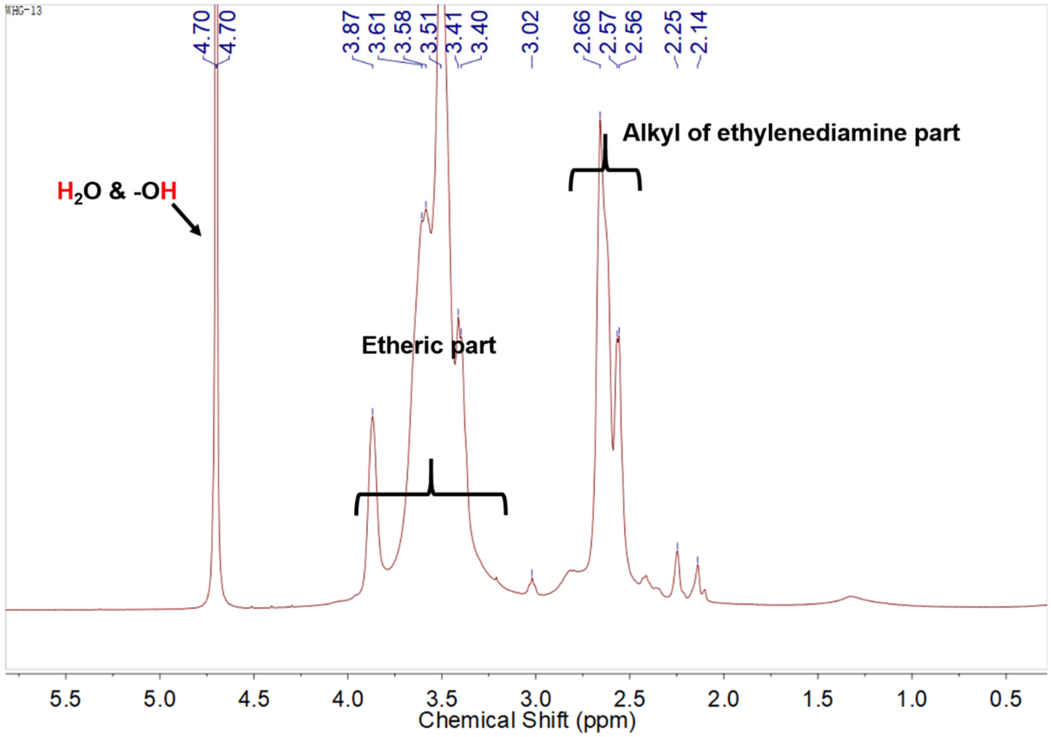

Figure S4. ^{1}H NMR spectrum of hbPGs-NH_2 in D_2O.

If the peak 4 in Figure 2 are attributed to the methenyl group which is near aldehyde group.

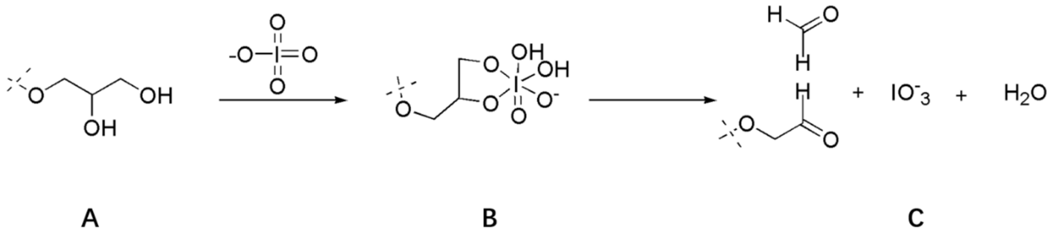

Figure 5. Reaction mechanism of vicinal diols oxidation using $NaIO_4$. A-C are the step for $NaIO_4$ oxidation process: A, original reactant; B, addition products with $NaIO_4$; C, final products.

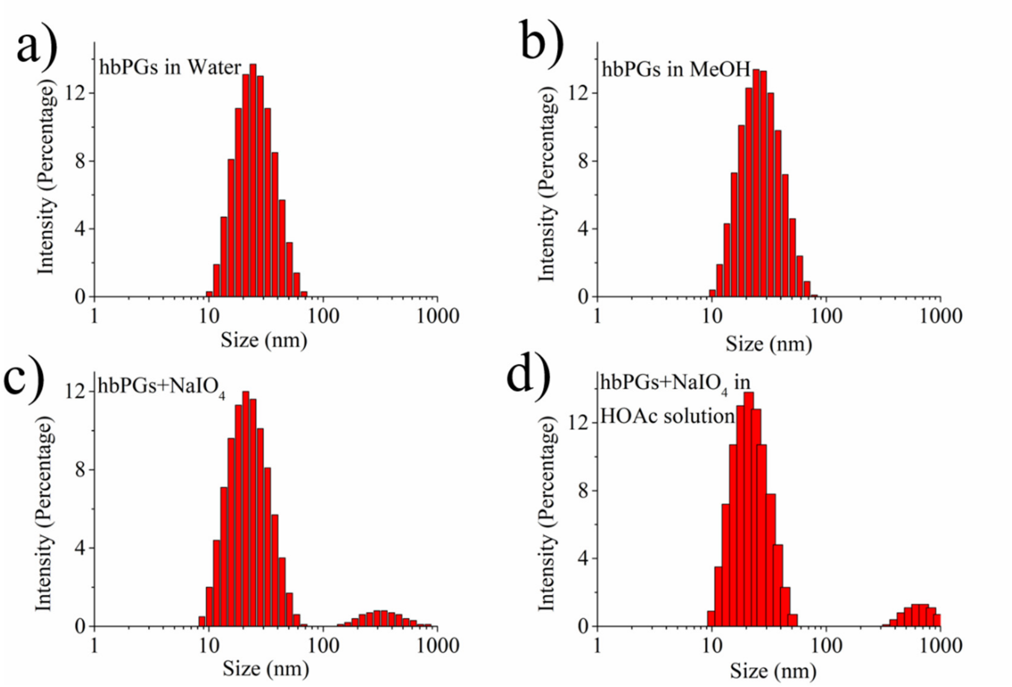

Figure S6. Hydrodynamic radius profile of (**a**) hbPGs in water, (**b**) hbPGs in MeOH, (**c**) hbPGs reacted with $NaIO_4$ (1.0 mg/mL) and (**d**) hbPGs reacted with $NaIO_4$ in HOAc solution (10% v/v aqueous solution).

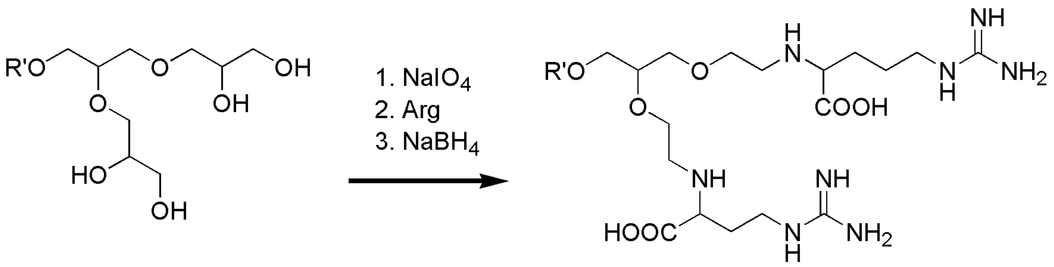

Figure S7. Synthesis route of Arg-tagged hbPGs.

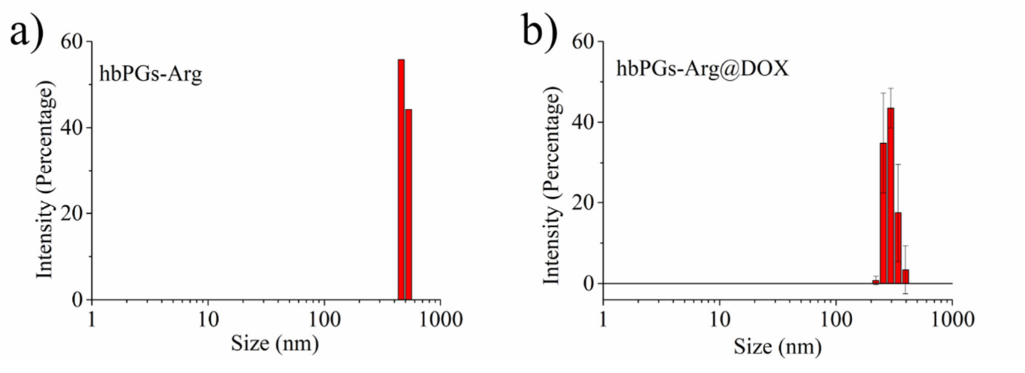

Figure S8. Hydrodynamic radius profile of hbPGs-Arg before (**a**) and after (**b**) encapsulating DOX molecules.

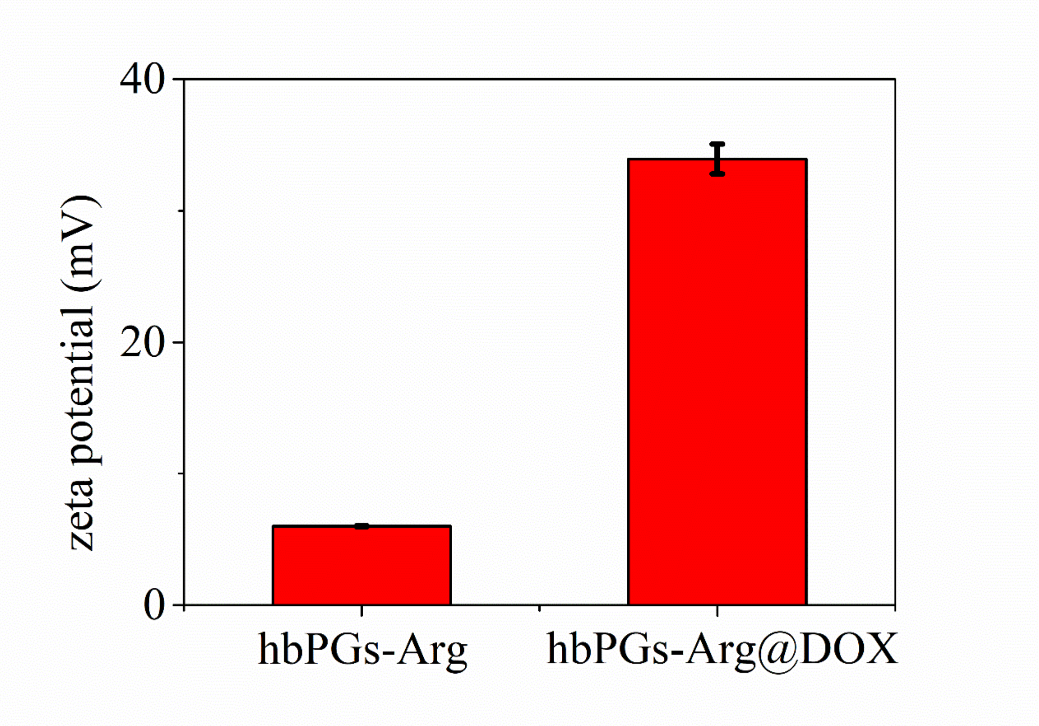

Figure S9. Zeta potential of hbPGs-Arg and hbPGs-Arg@DOX in aqueous solution (0.5 mg/mL for hbPGs-Arg).

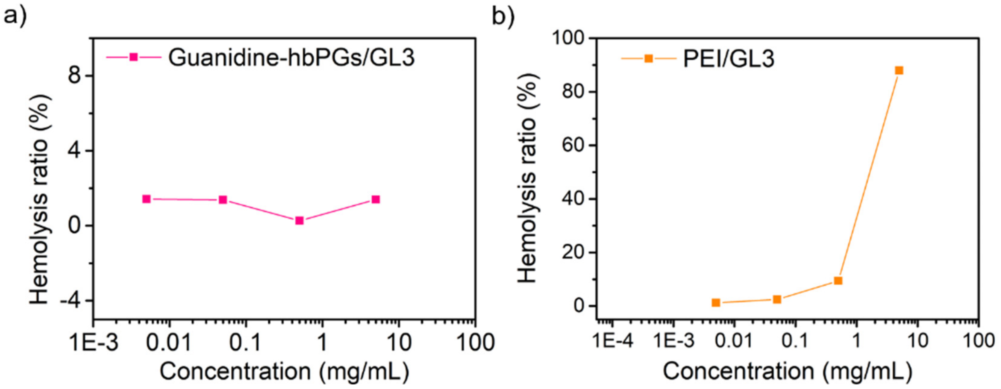

Figure S10. Hemolysis testing of guanidine-hbPGs (**a**) and negative control PEI (**b**). GL3 is the scrambled siRNA to drive the nanoparticle formation.

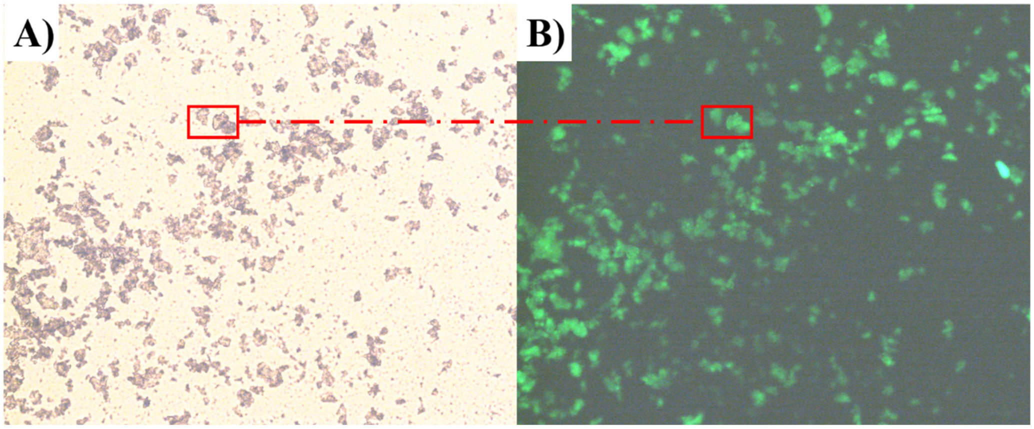

Figure S11. UCNP-*g*-hbPGs-GFP inverted fluorescence microscope in PBS solution **A**) bright field **B**) blue light emission field.

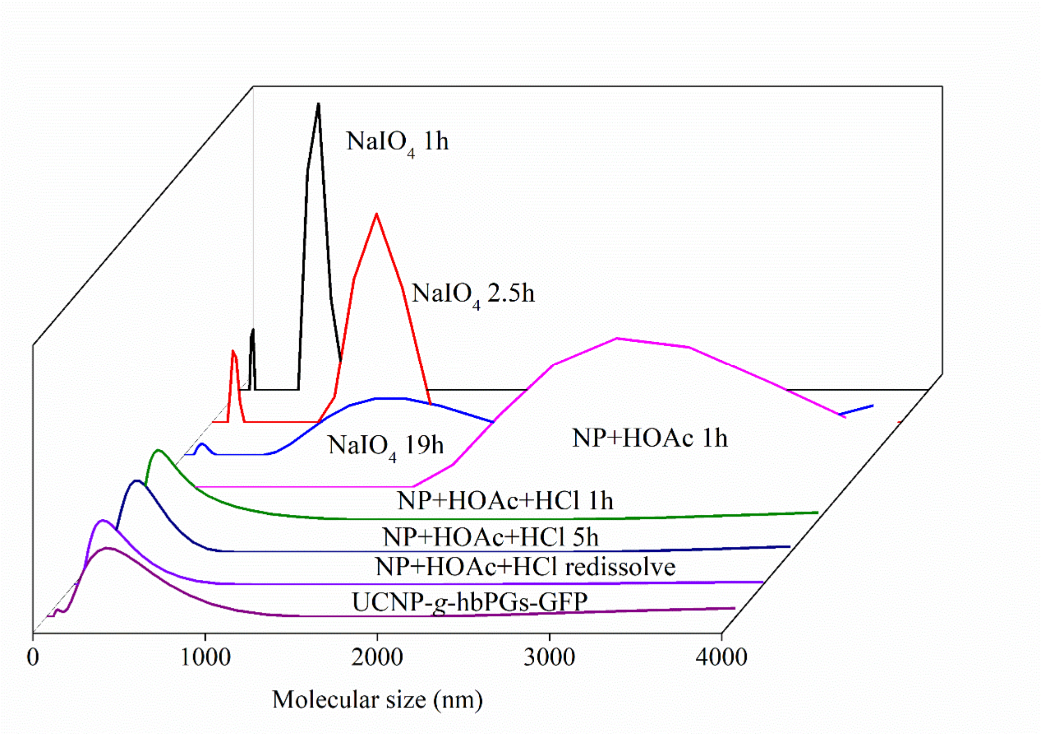

Figure S12. DLS size distribution of UCNP-*g*-hbPGs. Note: NP, UCNP-*g*-hbPGs; $NaIO_4$, dealt with $NaIO_4$.

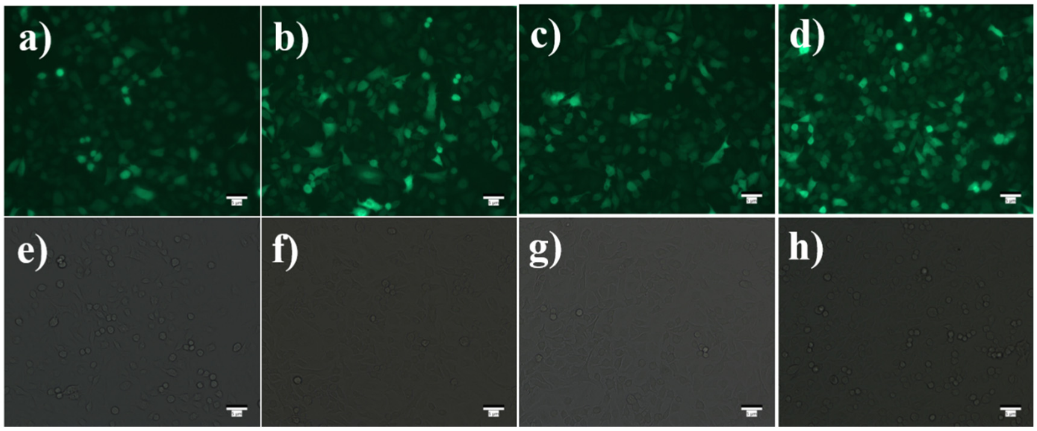

Figure S13. **a-d**) Fluorescence image of A549 cells incubated with different concentration GFP-g-UCNP solution. **e-h**). Bright field of A549 cells. The concentration from **a** to **d** is 10 μg/mL, 50 μg/mL, 100 μg/mL and 500 μg/mL. The white bar scale is 5 μm.

Publisher's Note: MDPI stays neutral with regard to jurisdictional claims in published maps and institutional affiliations.

© 2020 by the authors. Submitted for possible open access publication under the terms and conditions of the Creative Commons Attribution (CC BY) license (http://creativecommons.org/licenses/by/4.0/).
